# Application of artifact suppression algorithm of post-processing CBCT software in root canal filling materials

**DOI:** 10.1590/1807-3107bor-2025.vol39.011

**Published:** 2025-02-03

**Authors:** Carlos ESTRELA, Mônica Misaé Endo, Mike Reis Bueno, Bruno Correa Azevedo, Daniel Almeida Decurcio, Lucas Rodrigues Araujo Estrela

**Affiliations:** (a) Universidade Federal de Goiás - UFG, School of Dentistry, Department of Stomatologic Science, Goiânia, GO, Brazil.; (b) CROIF, Diagnostic Imaging Center, Cuiabá, MT, Brazil.; (c) OMFR Private Practice, Austin, TX, USA.; (d) Universidade Estadual Paulista – Unesp, Department of Endodontics, Araçatuba, SP, Brazil.

**Keywords:** Cone-Beam Computed Tomography, Endodontics, Diagnosis

## Abstract

Cone beam computed tomography (CBCT) is an imaging exam used increasingly in various fields of dentistry, and a greater number of endodontists are progressively gaining access to this technology. This study focused on applying an artifact suppression algorithm featured in CBCT software, and designed specifically to address artifacts related to root canal filling materials.

**Method:**

The sample consisted of eighty-four mandibular molars, with mesial root canals endodontically treated by using the lateral condensation technique. Four root canal sealers were applied: G1 – Sealapex®, G2 – AH Plus®, G3 – Endofill®, and G4 – Bio-C Sealer. CBCT scans were taken using PreXion 3D Elite®. Initially, the diameter of the root canal filling (in the mesiodistal and buccolingual directions) was measured using a digital micrometer (control). Next, these diameters were reevaluated in the CBCT images using the blooming artifact reduction (BAR) tool of the e-Vol DX software. The Van der Waerden nonparametric analysis of variance was performed, followed by applying the Tukey test to the normalized data. The significance level was set at α = 5%.

**Results:**

There were no statistically significant differences (p>0.05) in the measurement of original root canal filling materials obtained by the micrometer versus the e-Vol DX software in the mesiodistal and buccolingual directions.

**Conclusions:**

The tested software algorithm effectively suppressed artifacts resulting from obturation materials.

## Introduction

Cone beam computed tomography (CBCT) is an imaging exam used increasingly across various domains of dentistry, and a greater number of endodontists are progressively gaining greater access to this technology.^1,2,3,4,5^This examination has enhanced the efficacy of clinical diagnoses over the conventional 2-dimensional exams.^4^ CBCT has demonstrated greatly improved capabilities, particularly in complex conditions involving internal anatomy and proximity to critical areas, such as the detection of periapical lesions and root resorption.^4,5,6,7,8^ Its ability to navigate through various planes without interference from overlapping structures enables high-quality information,^9^ thus aiding in diagnosis and treatment planning, and making clinical decisions safer.^4,5,9,10,11,12^

Although CBCT offers numerous advantages in imaging, high-density materials such as solid or metal dental structures can introduce metal artifacts, thus compromising image quality and potentially leading to diagnostic inaccuracies.^13,14,15,16,17^ These artifacts manifest as distortions in CBCT images, by altering gray tones and fidelity to the actual structures, and ultimately diminishing image quality and hindering the diagnostic process. The beam hardening effect is caused by differential attenuation as the X-ray beams travel through high-density materials, resulting in various types of metal artifacts derived from beam hardening and scattered radiation.^3,13,14,15,16,17^

The confounding factors arising from loss of detail in CBCT images not only complicate interpretation, but can also obscure critical features, such as lines of root fractures, root perforations, root canal obturation materials, intraradicular posts, and other metal materials.^18,19,20,21,22,23,24,25,26^ Recently, a post-processing CBCT software (e-Vol DX)^18^ was developed, featuring specific algorithms designed to enhance image quality. This software has shown potential in reducing white artifacts^18,24,25^ on intraradicular posts made from various materials, a capability not yet demonstrated by other software solutions.

The e-Vol DX software was conceived to enhance the quality of images obtained by CBCT.^18^ This software provides high-resolution images with submillimetric voxel sizes that allow dynamic navigation across several image planes, and manipulation of volumetric parameters, such as thickness and slice intervals. Additionally, it offers features for data correction through image filters, as well as brightness and contrast adjustments. The advantages of this software include compatibility with all current CBCT scanners, and the ability to export data in DICOM format. Its brightness and contrast adjustments are more comprehensive, thus enabling a more thorough manipulation of the DICOM dynamic range, in comparation with other applications with limited adjustments. It also provides customized settings for slice thickness and sharpness, which are often restricted in other applications. The software incorporates an advanced noise reduction algorithm that improves image quality. In terms of resolution, the software allows screen captures at a resolution of 192 dpi, with an even higher option of 384 dpi, in contrast with the 96 dpi of similar applications.^18^

Root canal filling materials, such as gutta-percha and cements, consist of diverse chemical elements, each influencing different characteristics such as radiopacity, adhesion to root walls, and tissue tolerance.^27,28^ These materials often produce artifacts—such as light volume distortion, striations, and dark bands in CBCT images of endodontically treated root canals, owing to their composition of elements with varying molecular weights and high density.^19,20,21,23,24,25^ Studies have widely documented these artifacts as having the potential to introduce diagnostic errors in image interpretations. Consequently, several studies^18,19,20,21,23,24,25,26,29,30,31^ have aimed at mitigating these challenges to produce CBCT images that faithfully represent the real structures of the root canals, without inducing phantom images that could lead to diagnostic inaccuracies.

The demand for advanced technological resources to develop imaging exams that enhance the efficacy of diagnoses, treatment planning, clinical decision-making, and reliability and predictability of endodontic procedures, has spurred intensive research to provide sophisticated software solutions. The motivation for conducting this study stemmed from a gap in the existing research, notably the scarcity of studies leveraging specialized CBCT software algorithms designed to mitigate artifacts associated with root canal filling materials. Consequently, this study investigated the application of an artifact suppression algorithm in post-processing CBCT software specifically tailored to address artifacts related to root canal filling materials.

## Methods

### Sample selection

The raw (primary) data related to sample selection and preparation came from a previous study. Based on these primary data, the root canals were filled with several root canal sealers and then evaluated for volume discrepancy analysis according to the application of the artifact reduction algorithm.

This sample size was determined through a power analysis conducted using G*Power software version 3.1.2 (University of Düsseldorf, Germany), with an alpha error probability of 0.05 and 80% power (effect size = 0.5). The analysis indicated that a minimum sample size of 34 specimens was required. The present study used a sample of 84 mandibular molars that had been extracted for several reasons, and preserved in 0.1% thymol until ready for use. The teeth were then removed from the thymol solution and submerged in 5% sodium hypochlorite (NaOCl; Fitofarma, Lt. 20442, Goiânia, Brazil) for 30 minutes to eliminate external organic tissues.

CBCT scans were acquired to establish the inclusion criteria, wherein mandibular molars were chosen based on specific parameters comprising the presence of three canals, an intact pulp cavity, healthy root structure, complete root development, with a minimum length of 20 mm, and without internal or external resorptions, calcifications, abnormal tooth development, previous root canal treatment, or any history of orthodontic intervention. Approval for this study was obtained from the Institutional Ethics Committee (approval #06486919.0.0000.5083).

### Tooth preparation

The teeth were opened for root access, and all the root canals were instrumented and enlarged up to 400 μm using nickel-titanium (NiTi) engine-driven instruments. Patency was determined using a #15 K-file (Dentsply Maillefer, Ballaigues, Switzerland). The root canals were irrigated after each file change with 3 mL of freshly prepared 2.5% sodium hypochlorite solution to 2 mm short of the working length. Subsequently, the root canals were dried and treated with 17% EDTA (pH 7.2; Fórmula & Ação, São Paulo, Brazil) for 3 minutes to remove the smear layer. Afterward, the root canals were irrigated once again with 3 mL of 2.5% sodium hypochlorite solution, and dried using absorbent paper points.

After preparation, all the teeth were randomly divided into 4 groups (n=21) according to the following root canal sealers used for the experiments: Sealapex® (Sybron Endo, Orange, CA), AH Plus® (Dentsply, De Tray, Konstanz, Germany), Endofill® (Dentsply, Petrópolis, Brazil), and Bio-C Sealer (Angelus, Londrina, Brazil). Table 1 presents the composition of the materials under evaluation, as described by their manufacturers. The endodontic sealers were manipulated according to the recommendations provided by their respective manufacturers. Root canal filling was performed using the lateral condensation technique and calibrated gutta-percha points size 40.04 (Dentsply Maillefer, Sirona, Switzerland).

**Table 1:** Distribution of groups according to the materials used for root canal filling.

Group/Sealer	n = 84	Chemical Composition of Sealers	Manufacturer
G1. Sealapex^®^	n = 21	Base: N-ethyl toluene sulfonamide resin, fumed silica (silicon dioxide), zinc oxide, calcium oxide.	Sybron Endo, Orange, US
		Catalyst: isobutyl salicylate resin, fumed silica (silicon dioxide), bismuth trioxide, titanium dioxide pigment.	
G2. AH Plus^®^	n = 21	Paste A: bisphenol-A epoxy resin, bisphenol-F epoxy resin, calcium tungstate, zirconium oxide, silica, iron oxide pigments.	Dentsply, De Tray, Konstanz, Germany
		Paste B: dibenzylamine, aminoadamantane, tricyclodecane-diamine, calcium tungstate, zirconium oxide, silica, silicone oil.	
G3. Endofill^®^	n = 21	Powder: zinc oxide, hydrogenated resin, bismuth subcarbonate, barium sulfate, sodium borate.	Dentsply, Petrópolis, Brazil
		Liquid: eugenol, sweet almond oil.	
G4. Bio-C Sealer^®^	n = 21	Calcium silicates, calcium aluminate, calcium oxide, zirconium oxide, iron oxide, silicon dioxide, dispersing agent.	Angelus, Londrina, Brazil

### Measurement of root canal obturation with a micrometer

Root canal obturation was measured with a micrometer by stabilizing the mesial roots of the 84 specimens for cutting, and then fixing them with utility wax on an acrylic platform. The roots were cut mesiodistally at 6 mm from the root apex using a double-sided diamond disk (4” x 0.12 x 0.12, Extec, Enfield, USA) mounted on a microtome (Isomet 1000, Buehler, Bluff, USA) under water cooling, with the blade calibrated to 250 rpm. The root apex was separated from the mesiobuccal root by following the cut.

Subsequently, five small markings were made on the surface of each specimen using a carbide bur (FG 1/4 Kavo Burs®, Kavo Kerr, Joinville, Brazil) to establish the reference points. These points consisted of two on the X-axis (width), two on the Y-axis (height), and one on the Z-axis (depth). They served as standardized references for synchronizing the measurements taken with the digital micrometer (0-25 mm / 0.001 mm, Mitutoyo, Suzano, SP, Brazil). The micrometer was calibrated (certificate #07355/8) by the Metrology Laboratory of the Brazilian Calibration Network, following the pertinent standards (ABNT NBR/ISO IEC 170/25 CAL #0031, PML 0003). The measurements were aligned with corresponding images obtained from CBCT scans. The micrometer was positioned securely on a leveled prefabricated platform lifted to the position for taking measurements using a metal elevator.

The active tip of the digital micrometer (0.3 mm diameter) was aligned precisely with the specimens, by using the reference points of the buccolingual and mesiodistal markings. This alignment process was assisted by a surgical microscope (25X, Alliance, São Carlos, Brazil) to ensure the accuracy of the measurements. All the measurements were recorded in millimeters, on a thousandth scale, and documented meticulously in Excel spreadsheets for subsequent statistical analysis.

### Acquisition of the CBCT scan

The image acquisition followed a high-resolution protocol consistent with previous studies. The specimens in each group were distributed and mounted on two separate laboratory condensation silicone disks (Zetalabor®, Zhermack, Badia Polesine, Italy), each measuring 50 mm in diameter. One disk accommodated 11 specimens, while the other held 10. Subsequently, these bases with the specimens were immersed in a plastic container filled with water to simulate the attenuation of soft facial tissues.

Images were acquired in DICOM format using high-resolution tomography (PreXion, San Mateo, USA). The parameters for the 13-bit PreXion 3D Elite scanner included an isotropic voxel size of 0.100 mm, a field of view (FOV) measuring 52 mm × 56.00 mm (height by diameter), exposures lasting 33.5 seconds with 512 exposures per capture, X-ray output set at 90 kVp, a current of 4 mA, a focal spot measuring 0.20 mm × 0.20 mm, and a total beam filtration of >2.5 mm Al.

After image acquisition of the bases, and volume reconstruction using the PreXion 3D Elite, the DICOM files were processed using the following software package: e-Vol DX (CDT Software, São José dos Campos, Brazil). This package ran on a desktop computer equipped with Windows 10 (Microsoft Corporation, Redmond, WA, USA), featuring a 4.1-GHz i7-8750 processor (Intel Corporation, Santa Clara, USA), and an 8-GB NVIDIA GTX 1070 graphics card (NVIDIA Corporation, Santa Clara, USA). All the images were displayed and analyzed on a 27-inch P2719H monitor with a resolution of 1920 × 1080 pixels (DELL, Eldorado do Sul, Brazil).

Each specimen was first isolated from the others using the crop tool, and then aligned along the three anatomical planes – axial, coronal, and sagittal – to ensure that the sliced surface remained parallel to the ground. Final positioning of the specimens in the software was achieved by synchronizing them using five markings corresponding to the X-, Y-, and Z-axes. Additionally, CBCT scans were repositioned to correct possible parallax errors.

### Post-measurement on the CBCT scans

The methodology used in the present study has been previously employed in studies analyzing intraradicular posts.^24,25^ The dimensions of root canal fillings in CBCT images were measured by exporting the DICOM files acquired from each scanner with their original resolution, bit depth, and orientation, and subsequently imported into the e-Vol DX software. The measurements of all the CBCT images were obtained following the same positioning sequence. Each specimen was isolated from the others in the acquisition process, by using the “cut” tool, and then tilted in the three anatomical orientation planes (axial, coronal, and sagittal) so that the cut surface (slice) was parallel to the ground, oriented to correct the parallax bug. Synchronization of the 5 reference points marked on the specimens was used to achieve the final positioning of the specimen in the software. All the images obtained were viewed and analyzed using the same monitor described previously.

The specimen was positioned, and then the color map tool was applied. This tool displays grayscale images in various colors, representing the signal intensity of the material composing the specimens, as interpreted by the software. Root canal filling material appearing white or hyperdense was predominantly depicted in red. Subsequently, all initially red images underwent successive testing using the blooming artifact reduction (BAR) algorithm, with blooming checked across four different algorithm intensities. Each intensity level involved unique adjustments for brightness, contrast, enhancement, and dynamic range. The final validation included examining the grayscale image to visually confirm the outline of the object without any overlap of the hyperdense image on neighboring structures. The algorithm was selected based on color reformatting, to ensure that the peripheral area of the specimen under evaluation appeared in a color distinct from red.

After the initial evaluation, the BAR 1 algorithm was selected to analyze the specimens. Brightness, contrast, and enhancement levels were previously determined by the software. Owing to the hardening of rays and inherent radiation scattering in tomographic acquisition, two measurements were taken for each axis using the outermost point of the contour and the innermost point of the “blurring” from the hyperdense margin of the obturation material image.

The linear measurement tool was set to “millimeter,” the specimens were marked, and two measurement axes (mesiodistal and buccolingual) were traced on the post images. The measurement method involved determining the exact points to be measured, such as the markings on the edge of anatomical structures, and then adjusting the intermediate grade of the grayscale on the CBCT scan^32^. Initially, measurement of the correct positions was defined by identifying the point on the edge of the anatomical structure, and then adjusting the intermediate position on the grayscale of the CBCT image. Thin slices (0.10 mm) were then obtained from reconstructed 3D slices and measured by determining the edge of the anatomical surface in the axial plane. Replication of positions in 3D is done by using multiplanar reconstruction (MPR) of CBCT images using a positioning guide. The 3D density is adjusted to match the 2D image through calibration for alignment of 3D with 2D. This is performed only at the beginning of the alignment process. The intermediate position of the grayscale division is established in the CBCT scan. After completing one side, the process is repeated on the other side, using the 2D mode as a reference. Thus, the required measurements are obtained and determined at both points.^32^

Afterward, the intermediate grade of the grayscale was established on the CBCT scans. Once one side was completed, the guide was moved to the other side, and the same steps were replicated. The position of the marking was determined on the opposite edge using the 2D mode as a reference. The diameters of the posts were documented in an Excel spreadsheet for statistical analysis.

Two measurements were taken for each axis. Blooming artifacts can influence the image of the filling material due to beam hardening and radiation scattering, which are inherent to CT image acquisition. The references used were the outermost points of the contour of the post image (external bloom) and the internal points of the central and hyperdense contour of the root canal fillings. The average values were computed and logged in an Excel spreadsheet for statistical analysis (Table 2). All the measurements were performed by a radiologist and an endodontist, each with over 15 years of experience.

**Table 2:** Diameter of the root canal filling materials (G1 – Sealapex, G2 - AH Plus, G3 - Endofill, G4 - Bio C Sealer) using the micrometer and e-Vol DX software.

Group 1 Sealapex					Group 2 AH Plus					Group 3 Endofill					Group 4 Bio C Sealer				
Sample	Micrometer		e-Vol DX		Sample	Micrometer		e-Vol DX		Sample	Micrometer		e-Vol DX		Sample	Micrometer		e-Vol DX	
	BL	MD	BL	MD		BL	MD	BL	MD		BL	MD	BL	MD		BL	MD	BL	MD
1	0.914	0.995	0.914	0.996	1	0.889	0.735	0.889	0.737	1	0.756	0.689	0.758	0.691	1	0.716	0.690	0.718	0.691
2	0.811	0.784	0.812	0.784	2	0.694	0.744	0.694	0.744	2	0.739	0.755	0.741	0.757	2	0.664	0.704	0.665	0.707
3	0.784	0.782	0.784	0.785	3	0.660	0.676	0.660	0.676	3	0.662	0.649	0.668	0.658	3	0.682	0.702	0.683	0.704
4	0.656	0.686	0.656	0.687	4	0.743	0.720	0.745	0.724	4	0.684	0.698	0.688	0.704	4	0.984	0.695	0.988	0.695
5	0.906	0.722	0.907	0.722	5	0.692	0.686	0.695	0.687	5	0.709	0.732	0.712	0.736	5	1.185	0.796	1.187	0.799
6	0.812	0.780	0.814	0.781	6	0.678	0.795	0.679	0.796	6	0.730	0.748	0.730	0.749	6	0.579	0.622	0.581	0.624
7	0.654	0.726	0.654	0.727	7	0.756	0.856	0.756	0.856	7	0.749	0.786	0.751	0.788	7	0.675	0.730	0.677	0.731
8	0.887	0.960	0.887	0.962	8	0.776	0.667	0.778	0.668	8	0.761	0.846	0.761	0.847	8	0.710	0.806	0.711	0.809
9	0.679	0.720	0.680	0.720	9	1.063	0.976	1.065	0.977	9	0.753	0.765	0.753	0.765	9	0.739	0.658	0.742	0.660
10	0.725	0.704	0.726	0.704	10	0.649	0.717	0.651	0.718	10	0.783	0.755	0.783	0.755	10	0.750	0.693	0.752	0.696
11	2.412	0.472	2.449	0.475	11	0.698	0.726	0.700	0.729	11	1.410	0.860	1.414	0.864	11	1.025	0.685	1.029	0.687
12	0.744	0.823	0.744	0.823	12	0.844	0.760	0.850	0.761	12	0.681	0.731	0.682	0.732	12	0.736	0.753	0.737	0.756
13	0.847	0.771	0.847	0.773	13	1.011	0.731	1.013	0.732	13	0.634	0.658	0.636	0.666	13	1.060	0.741	1.062	0.741
14	0.667	0.726	0.667	0.726	14	1.071	0.940	1.075	0.943	14	0.690	0.703	0.691	0.703	14	1.470	0.747	1.475	0.749
15	1.690	0.786	1.693	0.791	15	0.807	0.641	0.807	0.641	15	0.738	0.716	0.739	0.718	15	0.694	0.642	0.696	0.645
16	0.691	0.691	0.691	0.692	16	1.185	0.740	1.189	0.742	16	0.713	0.661	0.715	0.663	16	0.785	0.744	0.786	0.744
17	0.709	0.738	0.710	0.739	17	0.695	0.795	0.697	0.804	17	0.802	0.908	0.803	0.908	17	0.744	0.638	0.749	0.642
18	0.829	0.824	0.829	0.824	18	1.794	0.842	1.800	0.844	18	2.885	0.413	2.895	0.416	18	3.828	0.760	3.832	0.762
19	1.079	0.429	1.084	0.437	19	0.827	0.634	0.830	0.637	19	0.828	0.620	0.831	0.624	19	0.727	0.710	0.728	0.710
20	2.070	0.760	2.095	0.763	20	0.680	0.702	0.683	0.706	20	0.730	0.699	0.738	0.704	20	1.001	0.798	1.003	0.800
21	3.723	0.764	3.737	0.767	21	0.770	0.773	0.773	0.774	21	3.948	0.662	3.959	0.664	21	0.580	0.604	0.584	0.606

BL: bucolingua/ MD: mesiodistal.

### Statistical analysis

The residuals of the statistical model showed heteroscedasticity and significant deviations from normal distribution. As a result, the Van der Waerden nonparametric analysis of variance was performed, followed by the Tukey test applied to normalized data. Intraobserver agreement was assessed using kappa statistics on 10% of the sample. The significance level was established at α = 5%.

## Results

**Figure 1:**
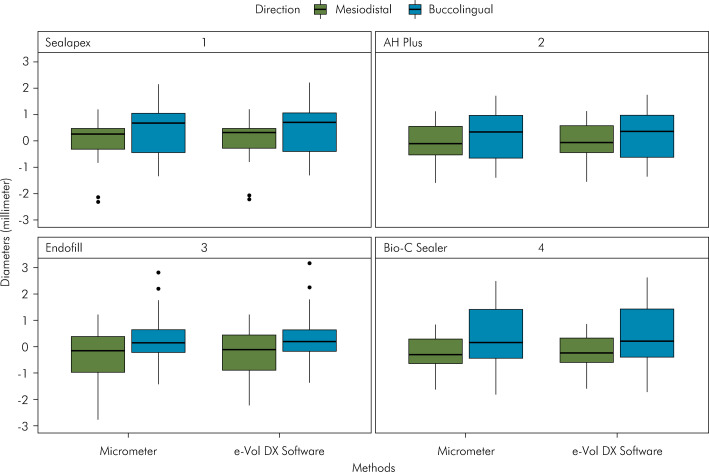
Values corresponding to measurements of the diameters of different types of filling materials, depending on the measuring instruments (micrometer and e-Vol DX software) and the directions (buccolingual, mesiodistal).

**Figure 2:**
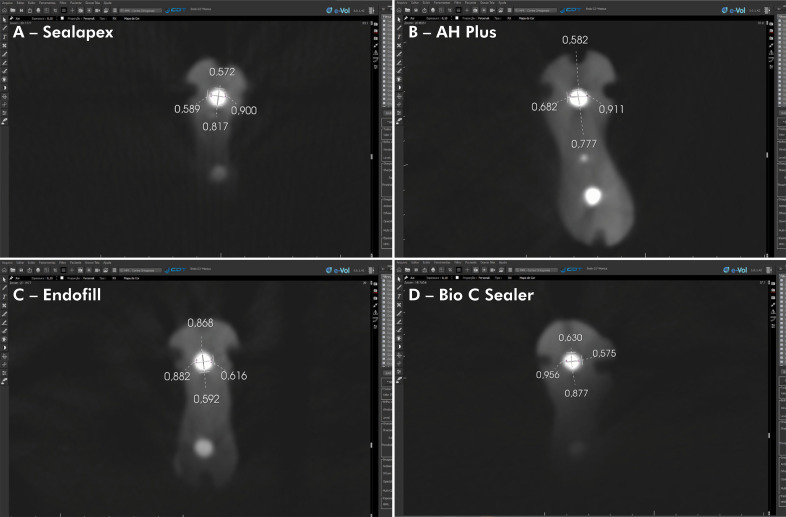
CBCT scans illustrate MD and BL measurements (X-axis, section/axial plane) using the outermost point of the contour of the post image, *i.e.*, the external blooming area, and the innermost point of the blooming artifact, which corresponds to the hyperdense margin of the post image.

Table 2 presents the raw values of the measurements of different filling materials using e-Vol DX software and a micrometer. Figure 1 shows the values of the filling material diameters in the mesiodistal and buccolingual directions performed by the CBCT software and the micrometer. According to the measurements made with the e-Vol DX software and the micrometer, the kappa values for interobserver agreement ranged from 0.86 to 0.96. There were no statistically significant differences in the measurements of root canal filling materials performed by the micrometer and the e-Vol DX software in the mesiodistal and buccolingual directions (p > 0.05) (Table 2; Figures 1 and 2).

Values represent MD and BL diameters (A) of Sealapex, (B) AH Plus, (C) Endofill, and (D) Bio-C Sealer using the e-Vol DX software.

## Discussion

There were no significant differences among the diameters of the root canal filling materials (Sealapex, AH Plus, EndoFill, Bio-C Sealer), even if we compare these differences using the measurements taken with the standard reference (control) of the digital micrometer. The artifact suppression algorithm of post-processing CBCT software (BAR) proved effective in reducing artifacts observed in CBCT scans of endodontically treated teeth.

Several filling materials were evaluated based on the root canal dimensions observed in the following CBCT scans: Sealapex (with calcium oxide), EndoFill (with zinc oxide and eugenol), AH Plus (epoxy resin with zirconia and tungsten), and Bio-C Sealer (a cement with several elements, especially tricalcium silicate, dicalcium silicate, and tricalcium aluminate). AH Plus stands out for its high radiopacity derived from zirconia and tungsten, whereas Sealapex originally had low radiopacity, but its formula was modified to include bismuth oxide, albeit in small quantities. EndoFill has adequate radiopacity derived from zinc oxide, as well as high levels of bismuth and barium. Bio-C Sealer, exhibits good radiopacity owing to zirconia oxide, calcium, and silica.^27,28,33^ These cements range in density due to their radiopacifying components and other additives, and their variations impair the quality of the CBCT images, potentially leading to artifacts.^20,21,22,34,35^

The artifacts are caused by root canal filling materials in the CBCT images, resulting from blooming, and substantially impact the quality of imaging examinations adversely, potentially obscuring fractures, root perforations, periapical or lateral rarefactions, etc. Application of the artifact suppression algorithm developed as a supplementary tool in the e-Vol DX software^18^ restores normal grayscale contrast in the image, preserving clarity by reducing the white areas in the original image. Consequently, the root canal filling materials cease to produce artifacts. This tool (BAR) allows adjustments of brightness, contrast, enhancement, and dynamic range at four different intensities (BAR 1, 2, 3, and 4). Final verification of the grayscale image confirms visual delineation of the object without invasion by a hyperdense image in the neighboring structures. In this study, endodontically treated teeth were analyzed using BAR 1 settings. Additionally, a color map was integrated into this tool, which can reformulate colors based on the molecular weight of the obturation material, thus ensuring that the peripheral area of the specimen retains a distinct color other than red^18^. This high-resolution capability enables the detailed observation of specific areas, such as lateral canals, perforations and root fractures, and the precise counting of gutta-percha cones in root canal obturations.^18^

Effective use of the e-Vol DX software hinges on understanding the fundamental principles of image capture, which are akin to those employed in photographic cameras.^18^ The RAW (native) image format encompasses the database of the captured object along with a significant number of pixels from the sensor. Several filters integrated into the e-Vol DX package are designed to comply with the principles of the RAW format, thereby preserving the quality of the acquired image and potentially recovering areas that may have been underexposed or overexposed. These filters serve to mitigate any loss in image quality, while enhancing the saturation and brightness of specific regions, particularly since luminous regions within the image consume substantial file space.^18^

Cone-beam image acquisition and reconstruction principles^3^ have shown that CBCT images can undergo various manipulations to enhance the visualization of anatomical structures and lesions after reconstruction. The most fundamental manipulation involves optimizing image contrast by displaying a selected portion of the full gray value range. This selected range uses the full contrast capabilities of both the display monitor and the human eye. These operations serve different purposes. In dental CBCT, they mostly enhance the contrast within the bone density range, corresponding to gray values indicative of bone tissue.^3^ Vasconcelos et al.^26^ assessed the effectiveness of two metal artifact reduction (MAR) algorithms in CBCT imaging, by evaluating various materials, positions of metal objects, and fields of view (FOVs). Nine phantoms containing cylinders made of amalgam, copper-aluminum (Cu-Al) alloy, and titanium were scanned using the Picasso Trio and ProMax 3D CBCT units with small and medium FOVs, both with and without MAR algorithms. Significant differences were found between the images with MAR and those without it for both CBCT devices. Amalgam exhibited the most pronounced artifact expression, followed by Cu-Al and titanium. Post-correction differences were observed only in Picasso Trio device images. The position of the metal cylinders within the FOV did not significantly impact the MAR algorithm performance in either CBCT device, but FOV settings did show significant differences in artifact expression, notably in ProMax 3D medium FOV scans after MAR correction. Overall, MAR algorithms effectively reduced artifacts, although their efficacy ranged according to the CBCT device, the FOV settings, and the metal object properties. Wanderley et al.^34^ investigated the impact of metal artifacts on image quality across 13 CBCT devices, and analyzed how scanning protocols and FOV influence the quality of CBCT images with and without metal artifacts. The study revealed a significant variation in image quality perception among the CBCT devices. Certain devices consistently outperformed others, regardless of the scanning protocol or clinical condition. Medium FOV standard scanning protocols received significantly higher ratings for detecting metal artifacts, whereas small FOV standard protocols performed better in the absence of metal. The subjective assessment of image quality ranges markedly across different CBCT devices and scanning protocols. Metal artifacts were found to substantially degrade image quality, with a considerable range in how well the devices could mitigate these artifacts. Selecting optimal protocols can partially reduce the impact of metal artifacts.

Although the focus of attention in this study was the artifact reduction algorithm of e-Vol DX software, another fundamental tool involved the method used for obtaining the measurements.^32^ In this context, some considerations about the applied methodology should be pointed out to enable a better understanding and the potential clinical application of these measurements, as follows: a) positioning accuracy: it is crucial to ensure precise positioning of the markings to obtain more reliable measurements – any inaccuracies in positioning could lead to errors in measurements; b) image calibration: calibrating the 3D density to match the 2D image ensures consistency and accuracy in measurements – this step is pivotal for achieving reliable and comparable results; c) reproducibility: the ability to replicate positions in 3D, and use of a positioning guide enhances the reproducibility of measurements – consistency in positioning is essential for obtaining reliable data across different scans and operators; d) determining intermediate positions: establishing the intermediate position of the grayscale division in the CBCT scan is crucial to ensure accurate delineation of anatomical structures; e) reference mode: using the 2D mode as a reference for repeating the process on the other side adds an additional layer of validation and consistency to the measurements. Understanding and optimizing these tools are essential for maximizing the utility of the methodology.

Several steps were taken to see through the methodology of the present study to ensure effective measurement of the obturation material and consistent positioning of the specimens in CBCT scans. This included standardizing root length, fabricating and stabilizing a platform with a metal elevator to ensure uniform micrometer use, mounting and stabilizing the specimens, marking the root with drills at three guide points (X-axis – width, Y-axis – height, Z-axis – depth) for subsequent synchronization, performing measurements at similar guide marks in the images of the obturation materials, and adjusting the parallax error.^18,23,24,25^

Ever since the CBCT was incorporated into endodontics, significant advancements have been made through studies to address the inconvenience of artifacts caused by solid high molecular weight structures.^13-25^ Volumetric distortion of root canal obturation materials has been observed in several studies,^18-26,29,35^ which use diverse methodologies to reduce this distortion in CBCT scans. Among the different techniques suggested to diminish metal artifacts, a map-reading strategy was proposed^19^ using CBCT to diagnose root perforations near metal intracanal posts. One such strategy to minimize the interference from metal artifacts involves acquiring sequential axial slices of each root, following a specific image navigation protocol from the coronal to the apical direction (or vice versa) with slices of 0.2 mm thickness. This approach enables dynamic visualization toward the area where the root canals communicate with the periodontal space, often associated with radiolucent areas indicative of root perforation. The map-reading approach helps resolve issues related to detecting root perforations near metal artifacts.^19^ Nevertheless, the final diagnosis should always combine clinical findings with imaging data.

The methodology of the present study was employed in previous studies analyzing intraradicular posts.^24,25^ The potential of blooming artifact reduction^24^ was assessed in human teeth prepared and obturated for intracanal post placement. The intracanal posts were distributed among anatomically customized prefabricated glass fiber posts, low-fusion alloy posts, and gold alloy posts. The results indicated that application of the BAR filter in the e-Vol DX software did not alter the dimensions of the intracanal posts in the CBCT scans, in comparison with the measurements made on the original posts taken with a micrometer. Another recent study^25^ evaluated the effectiveness of the PreXion3D® Image Analysis System and the e-Vol DX software in reducing blooming artifacts. This evaluation was conducted using the PreXion 3D Elite and the Carestream 9000C 3D scanners to examine teeth with intracanal posts. The findings demonstrated that employing the e-Vol DX CBCT software and DICOM images obtained from Carestream 9000C 3D and PreXion 3D Elite scanners effectively reduced blooming artifacts in images of metal structures.

The clinical relevance of the present study lies in its providing solutions to a significant issue associated with artifacts that lead to errors in CBCT image interpretations. Suppressing the artifacts produced by root canal obturation materials (such as cements and gutta-percha) in CBCT images ensures better clinical decision-making, enhances professional confidence, and improves predictability in questionable and complex clinical cases.

Further studies should focus on different brands of CBCT scanners, software variations, and new endodontic cements to better understand the processes involved in artifact formation and attenuation in CBCT images of endodontically treated teeth. These efforts aim to advance our understanding and optimize artifact management in clinical practice.

## Conclusions

No statistically significant differences were found in the measurements taken on original root specimens to determine root canal filling discrepancies, obtained by using either a digital micrometer or the artifact reduction algorithm of the e-Vol DX software. The tested software algorithm was capable of effectively suppressing artifacts from obturation materials.
